# MsDetector: toward a standard computational tool for DNA microsatellites detection

**DOI:** 10.1093/nar/gks881

**Published:** 2012-10-02

**Authors:** Hani Z. Girgis, Sergey L. Sheetlin

**Affiliations:** Computational Biology Branch, National Center for Biotechnology Information, National Library of Medicine, National Institutes of Health, 9600 Rockville Pike, Bethesda, MD 20896, USA

## Abstract

Microsatellites (MSs) are DNA regions consisting of repeated short motif(s). MSs are linked to several diseases and have important biomedical applications. Thus, researchers have developed several computational tools to detect MSs. However, the currently available tools require adjusting many parameters, or depend on a list of motifs or on a library of known MSs. Therefore, two laboratories analyzing the same sequence with the same computational tool may obtain different results due to the user-adjustable parameters. Recent studies have indicated the need for a standard computational tool for detecting MSs. To this end, we applied machine-learning algorithms to develop a tool called MsDetector. The system is based on a hidden Markov model and a general linear model. The user is not obligated to optimize the parameters of MsDetector. Neither a list of motifs nor a library of known MSs is required. MsDetector is memory- and time-efficient. We applied MsDetector to several species. MsDetector located the majority of MSs found by other widely used tools. In addition, MsDetector identified novel MSs. Furthermore, the system has a very low false-positive rate resulting in a precision of up to 99%. MsDetector is expected to produce consistent results across studies analyzing the same sequence.

## INTRODUCTION

Genomes contain a considerable number of repetitive elements known as repeats. These elements fall into two broad categories: (i) interspersed repeats or transposable elements and (ii) tandem repeats (TRs) (1). In this study, we focus on the detection of TRs. TRs occur as a result of replication slippage or DNA repair (2). Consecutive copies of a DNA motif comprise TRs. These copies can be exact copies in the case of perfect TRs or can be inexact copies in the case of approximate TRs. Depending on the length of the repeated motif, TRs can be classified as microsatellites (MSs) (the motif length is 1–6 bp) or minisatellites (the motif length is 10–60 bp).

MSs are important due to their documented functions and association with cancer and other diseases. In 2005, it was demonstrated that MSs polymorphism, which is due to copy number variability, can enhance the virulence of pathogens and their adaptability to the environment (2). In addition, MSs can be involved in gene regulation (3–5). Moreover, Kolpakov *et al.* (6) have highlighted several reported functions of MSs. Recombination enhancement has been linked to MSs consisting of a repeated GT motif (7). Further, alterations in dinucleotide MSs have been shown to be associated with cancer in the proximal colon (8). Trinucleotide MSs consisting of repeated CCG or AGC are associated with Fragile X syndrome, myotonic dystrophy, Kennedy’s disease and Huntington’s disease (9,10). Finally, several human triplet-repeat expansion diseases have been reported (11,12).

Furthermore, MSs have several biomedical applications. Ellegren (13) listed several applications of MSs in linkage mapping, population genetics studies, paternity testing and instances in forensic medicine. In the computational biology field, it is known that masking TRs in sequences improve the performance of sequence alignment methods (14).

Several computational tools have been developed to detect and discover repeats in DNA sequences. RepeatMasker (http://repeatmasker.org/) is a widely used detection tool, which searches a DNA sequence for instances of known repeats that have been previously identified. REPuter (15), PILER (16) and Repseek (17) are examples for *ab initio* discovery tools, which discover repeats classes in the input sequence without relying on a library of known repeats. In addition, special-purpose tools are available for the discovery and the detection of TRs/MSs in particular. STAR (18), Mreps (6) and Sputnik (http://espressosoftware.com/sputnik/index.html) are well-known MSs discovery tools. Hereafter, we use detection and discovery interchangeably. Several other tools are currently available (5,19–26). Additional tools are reviewed in (27,28).

However, these tools have the following limitations: (i) they require the user to adjust several parameters; (ii) the user may have to provide the filtering threshold(s) to remove spurious detections; (iii) some of the tools require a list of motifs or a library of known repeats and (iv) they may not be efficient in terms of memory or time. Two recent studies (28,29) have suggested that parameter tuning and the user-defined filtering threshold(s) result in varying the performance of these tools. Thus, based on the conclusions of these two studies, the need for a standard MSs detection tool is evident.

The goal of our study is to develop just such a tool to detect MSs in DNA sequences. To this end, we have designed software called MsDetector that attempts to remedy the limitations of the currently available tools. The parameters of our software tool were optimized using machine-learning algorithms. MsDetector does not require a library of known MSs or a list of motifs. Therefore, we expect MsDetector to produce consistent results across studies. In addition, MsDetector can process a whole human chromosome in a few minutes on a regular personal computer.

We incorporated a supervised-learning approach into our design. Labeled data are required for supervised-learning algorithms. For example, the labeled data required in our study to train a tool to detect MSs consisted of two sets of sequences: (i) DNA sequences that are known to include MSs and (ii) DNA sequences that are not likely to include MSs. To obtain such data, we used RepeatMasker to obtain MS sequences. Genomic sequences that did not overlap with MSs located by RepeatMasker comprised the other set unlikely to include MSs. Then, we trained a hidden Markov model (HMM) on these two sets to detect MSs. To reduce the false detection rate, the HMM detections were processed by a filter to remove spurious detections. Again, we applied a supervised-learning algorithm to obtain such a filter. We regarded the filtering problem as a classification problem where we distinguished between true and false detections. Therefore, we trained a general linear model (GLM) to obtain a classifier that functioned as the filter. As before, two sets of labeled data are required to train the filter. HMM detections that overlapped with MSs located by RepeatMasker comprised one of the two sets. The other set consisted of HMM detections found in shuffled DNA sequences. The human chromosome 20 and its shuffled version were divided into three segments to train, validate and test MsDetector. We followed the train–validate–test approach to make sure that MsDetector performance during training is very similar to its performance on unseen data, i.e. to avoid over-fitting.

MsDetector is both memory- and time-efficient. The memory requirement and the run time are linear with respect to the length of the input sequence. Due to the advantages of the supervised-learning algorithms, the user is not required to adjust any parameters or provide any filtering criteria. In sum, the contribution of our study comprises a software tool called MsDetector. The tool can locate perfect and approximate MSs. The advantages of MsDetector are as follows:

- The user is not required to optimize the parameters.
- There is no need to provide a library of known MSs.
- There is no need to specify motif patterns.
- It is efficient in terms of memory and time and
- It produces consistent results across studies.

## MATERIALS AND METHODS

### Overview

The goal of our work is to develop an easy-to-use computational tool that frees the user from optimizing several parameters. Therefore, we designed and developed a system we call MsDetector pronounced as m-s-detector. We assembled a pipeline of programs based on machine-learning algorithms to optimize the parameters of the tool automatically. The tool and the automated pipeline are available to the users (Supplementary Datasets 1–5). MsDetector consists of the following three components:

- Scoring component—a scheme to convert a series of nucleotides to a series of scores.
- Detection component—a two-state HMM to detect MSs.
- Filtering component—a GLM to remove false-positive detections.

We start by first defining the measures that were instrumental in the development of the tool. Then, we give the details of each of the three components.

### Evaluation measures

We used a collection of evaluation methods during the development of MsDetector. The sensitivity of tool *a* to MSs detected by tool *b* is measured as the percentage of the nucleotides located by tool *b* and also found by tool *a*. This measure is defined in Equation (1).
(1)
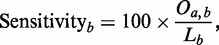

where *O_a,b_* is the length in base pairs (bp) of the overlapping segments of MSs detected by tool *a* and those detected by tool *b*. 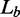 is the length of MSs detected by tool *b*.

To estimate the false-positive rate (FPR) of a tool, we run it on a shuffled version of the same sequence scanned by the tool. Nucleotides are shuffled independent of each other, i.e. a zero-order Markov model is assumed. The FPR measures the length of the false-positive detections in 1 Mbp of a shuffled DNA sequence. Equation (2) defines the FPR.
(2)
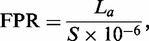

where *L_a_* is the length of MSs detected by tool *a* in a shuffled sequence. *S* is the length of the shuffled sequence. We estimated the FPR on a whole chromosome. A chromosome sequence usually includes the ‘N’ character. We shuffled the non-N regions only.

The precision measure tends to be used to calculate the ratio of true positives to false positives. However, since not all true positives are known, the standard precision definition has to be modified. We regard an MS detected by MsDetector as a true positive if it overlaps with an MS located by RepeatMasker. The rest of the MSs located by MsDetector are not necessarily false positives. However, we do not include them while calculating the precision. False-positive detections are those found by MsDetector in a shuffled version of the same sequence. Both the real and the shuffled sequences have the same length. The modified precision measure is defined according to Equation (3).
(3)
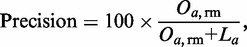

where 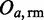 is the length (in bp) of the overlapping segments of MSs detected by RepeatMasker and MSs found by tool *a*. L*_a_* is defined as before.

The measures sensitivity*_b_*, the FPR and the precision depend on the detections of tool *a*. We do not add *a* as a subscript to simplify the notation.

In the rest of this section, we first discuss the data used to train the system. We then illustrate each of the three components in detail.

### Data

MsDetector is based on supervised-learning methods. Supervised-learning algorithms require examples and their labels. Therefore, to train MsDetector, we provided the algorithms with annotated sequences. Each nucleotide of these sequences was labeled according to its association with an MS region or a non-MS region. To obtain these labels, we used RepeatMasker to detect MSs in the training chromosome. A stand-alone version of RepeatMasker was used with the ‘-int -s -div -GC -species’ options. The -int parameter resulted in the extraction of simple repeats and low-complexity regions; other classes of repeats were not extracted. The value of the -GC parameter, which represents the GC content of the genome, was assigned 40 (different values were used according to the species). Only MSs that were deviated by at most 20% from the consensus sequence were reported. Detections that were deviated by >20% tended to be very degenerate. Hence, these detections could be a source of noise; therefore, they were not considered. Detections labeled as ‘simple repeats’ were extracted.

Three sets (training, validation and testing) were formed from the annotated chromosome. Using such sets while optimizing the parameters of a machine-learning algorithm is a classical approach to guard against over-fitting (30). Over-fitting occurs when the performance on the training set is excellent while the performance on unseen data is poor. Similar performances on the three sets indicate that there is no over-fitting. Traditionally, the algorithm is trained on one set, and the algorithm parameters are adjusted on the second set. Finally, the performance of the algorithm is tested on the third set. The performance on the testing set is a predictor of the future performance on new data. Each set included positive and negative sequences that were gathered from approximately one-third of the chromosome.

Next, we give the details of the scoring, the detection and the filtering components of MsDetector. We start with the scoring component.

### The scoring component

MSs are DNA sequences that are made of repeated words consisting of 1–6 nt. Given the nature of MSs, the flanking sequences of a certain word should include copies of this word. For example, the following sequence consists of 11 repeated ‘AT’ words, ATATATAT*ATATAT*ATATATAT. The flanking sequences of the middle word, the italicized *ATATAT*, include several copies of the same word. This concept comprises the underlying principle of the scoring component of MsDetector.

**Figure 1.**
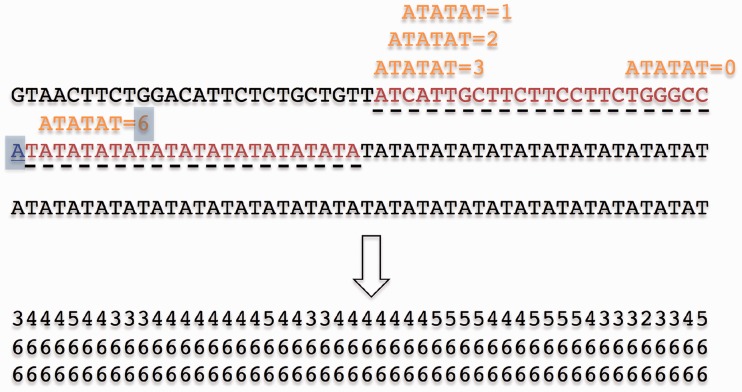
Converting a series of nucleotides to a series of scores. To score the nucleotide ‘A’ (surrounded by a gray box), we search for an exact copy or the best inexact copy of the word starting at ‘A’ within the flanking sequences (red with dashed underlines). Thus, we calculate the identity scores (Equation 4) of this word and every word in the left and the right flanking sequences. The score of the nucleotide ‘A’ is the best identity score. For example, the identity score of this word and the first word of the left flanking sequence is 3. The identity score of this word and the second word of the right flanking sequence is 6 which is the best possible score. Therefore, the score of the nucleotide ‘A’ is 6 (surrounded by a gray box). The score series, which is the output of the scoring component, is shown at the lower part of the figure. Notice the correspondence between the repeated ‘AT’ motif and the part of the output consisting of consecutive 6s.

The input of the scoring component is a series of nucleotides. It outputs a series of scores. To generate such a series, every nucleotide is considered to be the beginning of a word of length *n*. If an exact copy of the word is found in any of the two flanking windows, the score of this nucleotide is *n*. In other cases, the score of the best approximate match is assigned to this nucleotide. Specifically, to calculate the score of the *i*th nucleotide of a sequence, the word of length *n* starting at *i*, 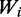, is aligned, without gaps, against the two sequences flanking the *i*th nucleotide (Figure 1). Let the length of each of the flanking sequences be *m*. We calculate the identity score of 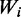 and the word 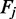 starting at nucleotide *j* of one of the flanking sequences, 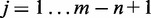. The score of nucleotide *i* is the best identity score of 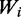 and all 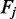. Next, we define the identity score of two words. Let *X* and *Y* be two subsequences representing two words of the same length: 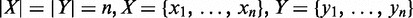. We define the identity score of *X* and *Y* as
(4)
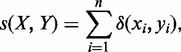

where
(5)
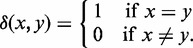

The running time of the scoring component is linear with respect to the length of the input sequence. Specifically, if the length of the input sequence is *h*, then the scoring component performs at most 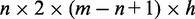 comparisons. Given that *m* and *n* are constants, the upper bound of the algorithm running time is *O*(*h*). The memory usage is also *O*(*h*).

The length of the word, *n*, is set to 6 bp. The maximum length of the repeated word, according to the definition of MSs, is 6 nt. This word length should be appropriate even if the repeated word is shorter or longer than 6 bp. In the case of a short motif, two or more repeated words should include a 6-bp repeated word. In the previous example, the length of the repeated word, AT, is 2 bp. Three subsequent ATs form a 6-bp word, ATATAT, which is also repeated several times in the sequence.

At this point, we have discussed the scoring component. We proceed by elaborating the detection component.

### The detection component

**Figure 2.**
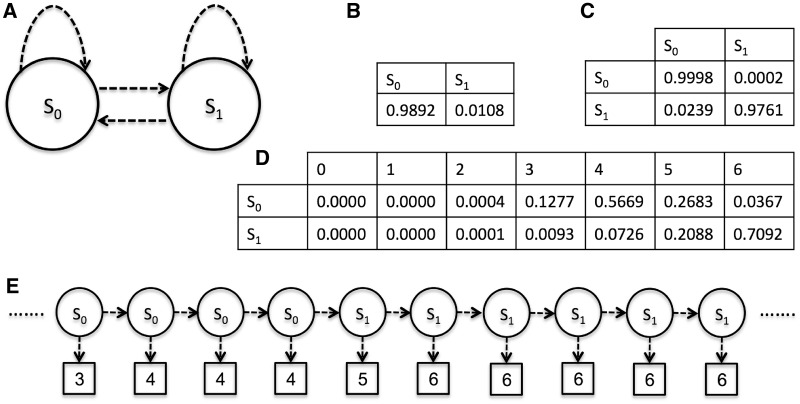
(**A**) The HMM structure. (**B**) The prior probabilities. (**C**) The transition probabilities. (**D**) The emission probabilities. (**E**) A series of states that likely generated a series of scores. 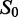 and 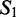 represent the non-MS and the MS states.

We developed a machine-learning approach to detect MSs. Our approach is based on a two-state HMM. The HMM was trained on a dataset which included sequences found in approximately one-third of the human chromosome 20. The scoring component was used to generate a series of scores representing the training portion of the chromosome. Then, this training portion was divided into 500-bp non-overlapping segments. HMMs are widely applied to time-series data. A series of scores can be considered as time-series data if we assume that a score depends on a few of the preceding scores in the series. We considered a DNA sequence to be made of MS regions and non-MS regions. Therefore, this two-state structure can represent a DNA sequence. The first state, 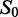, generates scores associated with non-MS regions which have lower scores, whereas the second state, 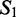, generates scores associated with MS regions which have higher scores. Generally, an HMM is described by three types of probabilities: prior, transition and emission probabilities (31). The priors are the probabilities that the series starts at one of the two states. The transition from one state to the next is described by the transition probabilities. State outputs, which are scores from 0 to 6, are represented by the emission probabilities. We used the training set to calculate the three types of probabilities. Figure 2 shows the HMM structure, the three types of probabilities and an example series of states that likely generated a series of scores.

Once the model is trained, the Viterbi algorithm can be used to find a series of states that likely generated the observed sequence of scores. Consequently, we applied the Viterbi algorithm to detect MS regions in DNA. We used the HMMlib (32) which provides a C++ implementation of the Viterbi algorithm. The run time of the Viterbi algorithm is linear with respect to the length of the input sequence.

One parameter that is likely to affect the HMM performance is the size of the search window. We varied the window size and studied the detections made by the HMM. The HMM was evaluated in terms of: (i) the sensitivity to MSs detected by RepeatMasker as defined by 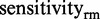 (Equation 1), (ii) the FPR (Equation 2) and (iii) the precision (Equation 3). To calculate the FPR and the precision of MsDetector, we used a shuffled version of the human chromosome 20. We shuffled the chromosome except the regions consisting of the ‘N’ character. Table 1 shows the performance of the HMM on the three sets. Using a window of size 24 bp resulted in slight over-fitting. The 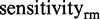 on the training set reached 88.9%, whereas the testing 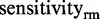 was 85.7%. In contrast, using longer windows resulted in more consistent performances across the training, the validation and the testing sets. As the window size increased, the FPR decreased. Next, we scrutinized the HMM to explain its behavior.

**Table 1.** The HMM performance on the three sets

Window	Training sensitivity (%)	Validation sensitivity (%)	Testing sensitivity (%)	Mean FPR (bp/Mbp)	Mean precision (%)
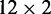	88.9	88.4	85.7	3827	70.8
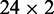	84.7	84.6	84.7	1481	85.7
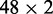	84.3	84.6	85.4	1324	87.0
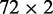	84.2	84.9	85.9	922	90.6
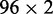	83.7	84.5	85.7	733	92.3

Sensitivity (Equation 1) is the percentage of the nucleotides of MSs detected by RepeatMasker and were also found by MsDetector. The mean of the FPRs (Equation 2) and the mean of the precisions (Equation 3) of MsDetector on the three sets are also shown.

**Figure 3.**
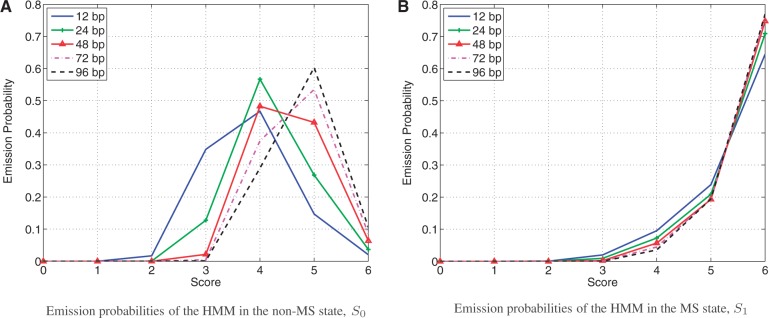
The effect of the length of the flanking sequences on the emission probabilities. We report the length of one of the two flanking sequences.

The score of a nucleotide in a sequence depends on the length of the search window. When the window length increases, the probability of finding an exact or a better approximate copy of the word also increases. We studied the emission probabilities obtained by using several window lengths. Figures 3A and 3B show the emission probabilities of the MS state and the non-MS state. From Figure 3B, it is possible to conclude that the window size has a minimal effect on the scores of the MS sequences, specifically if the length of each of the two flanking sequences is 24 bp or longer. In contrast, in the case of the non-MS sequences, when the window size increases, the probability of outputting higher scores also increases. Using a larger window complicates the detection of MSs because the scores of MSs may be similar to the scores of non-MS sequences. In addition, it is known that MSs cover a small percentage of the human genome, previously estimated as 3% (13). The small percentage of MSs in the human genome is captured by the prior probabilities of the HMM. The prior probabilities of a series of states to start in the non-MS state or the MS state are 0.9892 and 0.0108, respectively. Therefore, if the scores of the MSs and the non-MS sequences are similar due to a large search window, the HMM is more likely to be in the non-MS state than in the MS state. In other words, as the window size increases, the HMM becomes less sensitive for detecting MSs resulting in lower FPR.

Recall that MsDetector consists of three components. We have discussed the scoring and the detection components. We continue by giving the details of the filtering component.

### The filtering component

The purpose of this component is to remove detections that are similar to those found in random sequences. To this end, we designed a machine-learning approach to process the detections of the HMM. We represented a detection, consisting of a series of scores, in terms of two features: the length of the detection and its average score. The average score is the sum of the scores in the series divided by the length of the series. Long detections that have high average scores are likely to be true MSs. Short detections that have low average scores are likely to be false positives. Combining these two features can provide a powerful method to remove undesired detections. The two features are not equal in terms of their effectiveness in removing erroneous detections. Therefore, we needed to determine the weight associated with each of the two features. The task at hand can be formulated as a classification problem where the goal is to find the best weights that can separate the positive detections from the negative ones. To this end, we trained a GLM (33) on a labeled dataset to find the optimal weights of the two features. Positive and negative labels were assigned as follows: (i) HMM detections that overlapped with RepeatMasker MSs were considered positives (labeled by 1) and (ii) detections that were detected in the shuffled sequence were considered negatives (labeled by −1). To generate the shuffled sequence, the independence of the nucleotides was assumed. Therefore, the shuffled sequence had the same mono-nucleotide composition as the original sequence. Similar to the dataset used to develop the detection component, this labeled dataset was divided into three sections for training, validation and testing.

Normalizing the data is usually recommended before applying the optimization algorithm, i.e. before fitting the model (30). There are several methods to normalize the data. In this work, we applied the optimization algorithm to the *z*-scores of the features instead of the features themselves. Equation (6) illustrates the normalization step.
(6)
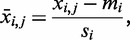

where 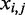 is the *i*th feature of an HMM detection *j* (*i* = 1, 2); 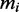 and 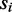 are the mean and the standard deviation of the *i*th feature of HMM detections in the training set. The mean and the standard deviation of the lengths were 25.537 and 35.802. The mean and the standard deviation of the average scores were 5.7773 and 0.23168.

Equation (7) gives the form of the solution found by the GLM.
(7)
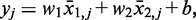

here, 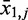 and 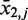 are the *z*-scores of the features of an HMM detection *j*; 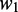 and 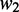 are the weights associated with the *z*-scores of the two features of *j*; *b* is the error; 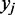 is the label; We used a Matlab implementation of the GLM with a logistic activation function (33). The optimization algorithm converged in 10 iterations at most. The logistic function (Equation 8) was applied to the linear combination as defined by Equation (7). Detections with logistic values 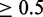 are considered MSs.
(8)
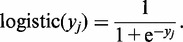



We varied the window size while evaluating the HMM combined with the GLM-based filter. Table 2 shows the results on the three sets. By comparing these results to the ones obtained without the filter (Table 1), the effectiveness of the GLM-based filter was proven. The GLM-based filter was able to reduce the FPR dramatically, while maintaining high 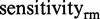. Consequently, the precision of the system approached 100% compared with a precision of 71–92% obtained without the filter. These results show that the size of the window has a minimal effect on the performance of the full system. However, the performance based on a half window size of 12 bp indicated slight over-fitting manifested by higher training 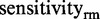 of 87.3% and lower testing 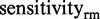 of 83.2%. We decided to use a half window size of 24 bp as the default of the distribution version of MsDetector due to two factors. First, a smaller window size leads to a better execution time. Second, this window size resulted in consistent sensitivities across the three sets.

**Table 2.** The performance of the HMM combined with a GLM-based filter

Window	Training sensitivity (%)	Validation sensitivity (%)	Testing sensitivity (%)	Mean FPR (bp/Mbp)	Mean precision (%)
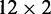	87.3	86.5	83.2	43	99.5
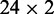	83.4	83.4	83.4	40	99.5
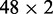	83.0	83.6	84.1	36	99.6
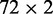	83.0	83.8	84.7	39	99.6
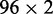	82.5	83.4	84.6	41	99.5

The size of the window is shown under column ‘Window.’ The sensitivity, FPR and precision are defined in Equations (1–3). The sensitivity is calculated with respect to the detections by RepeatMasker. The average FPR and the average precision of MsDetector on the three datasets are reported in the last two columns.

**Figure 4.**
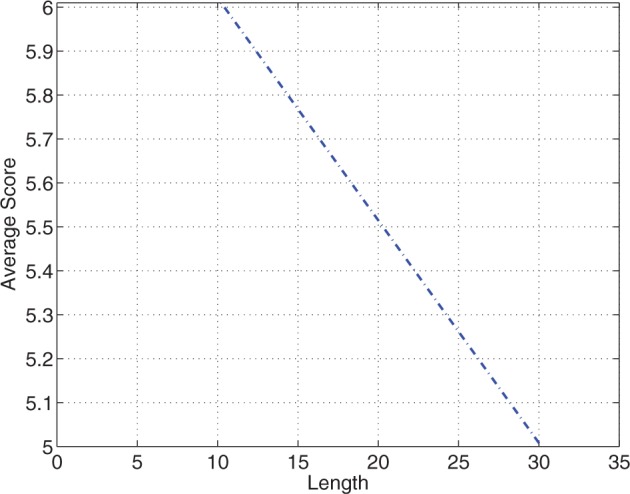
The linear function representing the GLM-based filter. HMM detections that have lengths and average scores below the line are considered negatives. On the other hand, detections that have lengths and average scores on or above the line are considered positives.

The final MsDetector filter is based on the solution found by the optimization algorithm. Equation (9) shows the weights associated with the *z*-scores of the features.
(9)
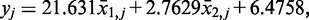

where 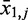 is the *z*-score of the length of detection *j*; 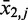 is the *z*-score of the average score of detection *j*. The weight associated with the length is greater than the weight associated with the average score indicating that the length is a more important filtering criterion. Figure 4 shows a line specifying the filtering function.

In sum, we developed MsDetector to locate MSs in DNA sequences. The parameters of MsDetector were optimized on the human chromosome 20. MsDetector is easy to use, only requiring an input sequence(s) in FASTA format. The output of MsDetector can be in two formats. The first format is the masked sequence in FASTA format. The detected MSs are marked by lower case letters and the rest of the sequence is written in upper case letters. The second format is the genomic locations of the detected MSs and their logistic values.

In the next section, we evaluate MsDetector on chromosomes from the human and other five species. We also compare the performance of MsDetector with the performances of three related and widely used tools.

## RESULTS

Our study resulted in the software that we call MsDetector. The user is not burdened by having to optimize the parameters of the software; we optimized the parameters by applying machine-learning algorithms to one of the human chromosomes. MsDetector, although optimized on the human chromosome, can be applied to genomes of other species successfully. In addition, we provide a pipeline to automatically optimize the parameters on a chromosome of a species of interest to the user. The pipeline requires the sequence of the chromosome and a list of MSs detected by RepeatMasker. MsDetector is easy to use: the user only needs to provide MsDetector with the input sequence(s) in FASTA format.

### Software availability

The software is available as Supplementary Datasets 1–3. The C++ source code is included in Supplementary Dataset 4. Supplementary Dataset 5 includes the automated training pipeline. MsDetector and the training pipeline can be found at http://www.ncbi.nlm.nih.gov/CBBresearch/Spouge/html_ncbi/html/index/software.html.

### Evaluation

In addition to the three measures explained in the ‘Materials and Methods’ section, we used two additional criteria to evaluate MsDetector and the other tools. These criteria comprise the percentage predicted (PP) and the execution time. The PP is the percentage of the length of the chromosome predicted as MSs. Equation (10) defines the PP:
(10)
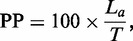

where *L_a_* is the length of MSs detected by tool *a*. *T* is the length of the scanned chromosome. We consistently used computers with the same specifications to measure the execution time of the tools. Specifically, all tests were performed on computers with 2 Intel Xeon 6 cores 2.93 GHz CPUs and 48 G RAM. CentOS 5.6 x86_64 is the operating system installed on all the computers.

In sum, the evaluations were conducted to focus on (i) the sensitivity to MSs detected by widely used methods such as RepeatMasker and STAR, as per Equation (1); (ii) the FPR, as per Equation (2); (iii) the precision, as per Equation (3); (iv) the PP, as per Equation (10) and (v) the execution time of the tools.

As noted in (29), the outputs of MSs detection/discovery tools vary considerably due to the user-adjustable parameters. The available computational tools have the potential to detect MSs accurately; however, only experienced users can obtain such results. For example, the methodologies of TRF (34) and Tantan (14) are similar to that of MsDetector. Given a set of parameters, the performance of TRF and Tantan can be very comparable to that of MsDetector. However, the user is required to adjust several parameters and to develop evaluation measures to find the set of parameters that result in the best performance of TRF or Tantan. These two tasks can be too difficult for a novice user. The main goal of our study is to produce an easy-to-use software with optimized parameters. Consequently, the user does not need to calibrate the tool. To evaluate the success of our efforts, we compared MsDetector with other tools that can be executed in the default mode, i.e. a non-expert user can run the tool without adjusting the parameters. Similar evaluation method was used in (35).

### Results on chromosomes from the human, *Drosophila melanogaster*, *Arabidopsis thaliana* and *Saccharomyces cerevisiae*

We trained, validated and tested MsDetector on sequences from or based on the human chromosome 20. MsDetector was compared with STAR, Mreps and Tantan on four chromosomes from the human, *D. melanogaster*, *A. thaliana* and *S. cerevisiae*. STAR does not require adjustable parameters; however, it requires a set of motifs. As recommended by the inventors of STAR, we used a set of 964 Lyndon motifs which are 1–6 bp long. If the chromosome was large, we ran STAR on 1-Mbp-long fragments due to the long processing time STAR required. We used the Mreps software in the default mode, i.e. we did not provide the value of the resolution parameter. Similarly, the default options of Tantan were used. Table 3 shows the performances of the four tools. The performance patterns of the tools were very similar on the different chromosomes. In sum, we made the following five observations:

- Tantan achieved very high sensitivity to MSs detected by RepeatMasker. However, it has the highest FPR resulting in the lowest precision.
- The performance of Mreps was moderate in general.
- STAR consistently achieved high sensitivity to MSs detected by RepeatMasker, the lowest FPR and the highest precision. This excellent performance came at the price of long execution time.
- MsDetector achieved high sensitivity to MSs located by RepeatMasker. Its FPR and precision were consistently the second best after those achieved by STAR. MsDetector is time-efficient in comparison to STAR.
- The results on the non-human chromosomes show that the performance of the default version of MsDetector, trained on the human chromosome 20, is comparable to that of a version trained on a chromosome of the same non-human species. The species-specific HMMs and GLMs are available as Supplementary Dataset 6.

**Table 3.** Tools performance on different species

Tool	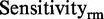 (%)	FPR (bp/Mbp)	Precision (%)	PP (%)	Time (s)
Human chromosome 19 (59.1-Mbp long)					
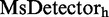	83.3	34	99.7	3.0	29
STAR	94.7	10	99.9	3.1	49 588
Mreps	70.8	346	97.0	2.1	15
Tantan	92.8	2842	83.6	8.4	43
*D. melanogaster* chromosome 4 (1.4-Mbp long)					
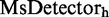	89.0	161	98.2	2.6	1
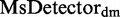	93.6	520	94.7	3.7	1
STAR	92.6	7	99.9	1.6	1284
Mreps	71.7	860	89.2	2.1	1
Tantan	94.0	9538	49.4	7.6	2
*A. thaliana* chromosome 3 (23.5-Mbp long)					
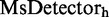	74.9	214	90.8	1.4	12
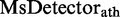	73.8	67	96.8	1.1	13
STAR	87.6	48	98.1	0.7	21 793
Mreps	67.7	817	70.0	1.30	7
Tantan	90.7	8479	23.1	7.30	17
*S. cerevisiae* chromosome 7 (1.1-Mbp long)					
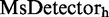	74.1	174	92.5	0.8	1
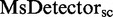	81.7	130	94.8	1.0	1
STAR	88.4	6	99.8	0.7	1019
Mreps	67.3	741	72.5	0.9	1
Tantan	90.0	6922	27.4	3.6	1
*P. falciparum* chromosome 7 (1.5-Mbp long)					
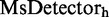	81.7	2945	97.2	21.9	2
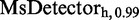	78.0	965	99.0	19.6	2
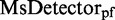	75.2	896	99.0	17.0	2
STAR	96.5	64	99.9	28.3	2025
Mreps	63.3	3518	96.4	13.6	1
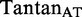	67.4	1434	98.6	15.9	3
*M. tuberculosis* circular chromosome (4.4-Mbp long)					
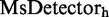	54.7	250	70.0	0.8	3
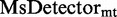	76.6	39	95.4	2.0	3
STAR	88.8	4	99.6	1.0	2959
Mreps	21.9	961	19.7	0.7	1
Tantan	88.1	10 270	8.4	5.7	4

Column ‘
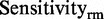
’ displays the percentage of the nucleotides that were detected by RepeatMasker as MSs and were also detected by one of the four tools (Equation 1). FPR is the false-positive rate (Equation 2). Precision is defined by Equation (3). PP is the percentage of the chromosome predicted as MSs (Equation 10). The time that a tool took to process the chromosome is reported under ‘Time.’ 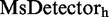 was trained on the human chromosome 20; the threshold of the GLM was 0.5. 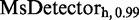 was trained on the same chromosome; however, the threshold of the GLM was 0.99. 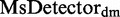, 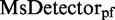, 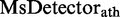, 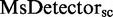 and 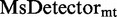 were trained on one-third of the *D. melanogaster* chromosome 3R, *P. falciparum* chromosome 14, *A. thaliana* chromosome 5, *S. cerevisiae* chromosome 4 and *M. tuberculosis* circular chromosome, respectively. We used a half window of size 24 bp for all models except the model of 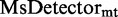, for which we used a half window of size 48 bp. The parameters of 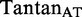 were the ones recommended by the author for AT-rich genomes. Specifically, we used the ‘atMask’ scoring matrix and the value of the parameter ‘*r*’ was assigned 0.01. All other parameters were the defaults.

These results demonstrate the capability of MsDetector to mine for MSs in the human genome in addition to genomes of other species including insects, plants and yeast.

### Results on the human genome

MsDetector was used to locate MSs in the human genome. The genomic locations of the detected MSs are available as Supplementary Dataset 7. MSs found by MsDetector comprised ∼1.6–3.0% of each chromosome. The sensitivity to RepeatMasker detections ranged from 80.3 to 83.7%. MsDetector achieved a consistently low FPR of 22–136 bp/Mbp. The precision of MsDetector reached 99.7%. Overall, the total length of MSs located by MsDetector represented 1.95% of the human genome. The FPR of MsDetector on the human genome was 81 bp/Mbp. These results demonstrate the success of MsDetector to detect MSs in the human genome.

### Results on the *Plasmodium falciparum* chromosome 7

The *P. falciparum* (malaria) has the most AT-rich known genome (∼80%). Detecting MSs in such a genome is a challenge. Further, evaluating a computational tool on this genome represents another challenge, specifically, evaluating its FPR. Due to the high AT content of this genome, shuffling one of its chromosomes is likely to result accidentally in repetitive sequences resembling MSs. To circumvent this problem, we used RepeatMasker to search for MSs and low-complexity regions in the shuffled chromosome. While calculating the FPR of a tool, detections that were included in the MSs or in the low-complexity regions were not considered false positives. Recall that the FPR is calculated on the shuffled chromosome.

We started by evaluating the default version, 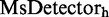, trained on one-third of the human chromosome 20, on the malaria chromosome 7. 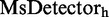 attained high sensitivity to RepeatMasker detections (81.7%) and high precision (97.2%) while the FPR reached 2945 bp/Mbp and the percentage of the chromosome predicted as MSs (PP) reached 21.9%. Although the precision of 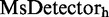 was very high, it did not result in similar FPR or PP on the chromosomes tested from other species. Similarly, the PP obtained by RepeatMasker on this chromosome (12.3%) was much higher than what was observed in other species (0.1–1.6%). Given the unusual nucleotide composition of this genome, we decided to consider MsDetector detections that are more likely to be true positives. To this end, we increased the threshold of the filter to 0.99 which is nearly the maximum output of the logistic function. We call this version 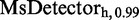. Recall that the default threshold of the filter is 0.5, i.e. if the output of the logistic function is ≥0.5, the detection is considered positive. Similarly, the author of Tantan designed a special scoring matrix to handle the AT-rich genomes.

The performance of 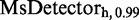 on the malaria chromosome confirmed the previous results on the other species (Table 3). 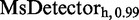 achieved the second highest sensitivity to RepeatMasker detections, the second lowest FPR and the second best precision. In contrast, STAR attained the highest sensitivity, the lowest FPR and the best precision. However, MsDetector is much faster than STAR. The performance of 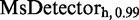 and the performance of a version trained on another malaria chromosome were similar. In sum, these results demonstrate that MsDetector can locate MSs in genomes with unusual nucleotide composition.

### Results on the *Mycobacterium tuberculosis* genome

The genome of the *M. tuberculosis* CDC1551 strain (a pathogenic bacteria) consists of one circular chromosome. Repbase, the library used by RepeatMakser, does not include simple repeats specific to bacteria or to prokaryotes in general. To calculate the sensitivity to MSs located by RepeatMasker, we specified the ‘species’ option of RepeatMasker as eukaryota. Therefore, we should consider this fact as well as the small number of MS loci detected by RepeatMasker (67 loci) while analyzing the sensitivity. Again, the default version of MsDetector came second after STAR in terms of the sensitivity to RepeatMasker detections, the FPR and the precision (Table 3). However, MsDetector was much faster than STAR. Even though MsDetector achieved the second best sensitivity, its sensitivity was low (∼55%) in comparison to its performance on the chromosomes of the other five species. A version of MsDetector that is trained on one-third of the *M. tuberculosis* chromosome attained higher sensitivity, ∼77%. The overall performance of this version was comparable to that of STAR. Based on these results, MsDetector can be used to detect MSs efficiently and accurately in bacterial genomes.

### MsDetector sensitivity to STAR detections

We have reported the sensitivities of MsDetector to MSs located by RepeatMasker in the previous experiments. As MsDetector was trained on MSs found by RepeatMasker, the high sensitivity of MsDetector to RepeatMasker detections is expected. The excellent precision of STAR motivated us to analyze the sensitivity of MsDetector to MSs identified by STAR, 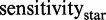 (Equation 1). We found that MsDetector achieved high 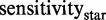. Specifically, the 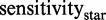 of MsDetector on the human chromosome 19, the fruit fly chromosome 4, the *A. thaliana* chromosome 3, the yeast chromosome 7, the malaria chromosome 7 and the genome of *M. tuberculosis* were 70.7, 80.1, 56.2, 63.1, 63.6 and 47.2%, respectively. These results show that MsDetector is sensitive to MSs found by STAR, even though MsDetector was trained on MSs located by RepeatMasker.

### Identification of new MSs by MsDetector

The ability of a tool to detect new repeats is one of the criteria Lerat (1) has used to evaluate several repeats-finding programs. Consequently, we evaluated the ability of MsDetector to locate new MSs that were not identified by either RepeatMasker or STAR. We studied the MSs identified by MsDetector in the human chromosome 19. Approximately 75% of the MSs located by MsDetector overlapped with MSs found by RepeatMasker or STAR or both. These results show that 25% of the MS loci detected by MsDetector were uniquely identified by MsDetector. Table 4 provides examples of these MSs. Strand slippage, one of the mechanisms responsible for generating MSs, is likely to occur in the sequences shown in the table due to their repetitive structure. In general, we observed that approximate copies, rather than exact copies, of a motif(s) comprised these sequences. The long repeated motifs (∼20 bp) of the last two sequences in Table 4 suggest that these sequences are minisatellites.

**Table 4.** Examples of MSs located by MsDetector but missed by RepeatMasker or STAR or both

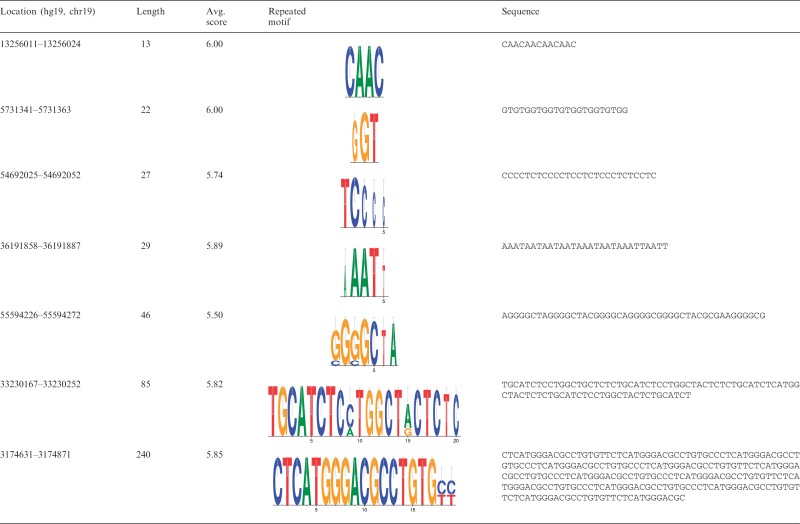

Motif logos were generated by WebLogo (36).

**Figure 5.**
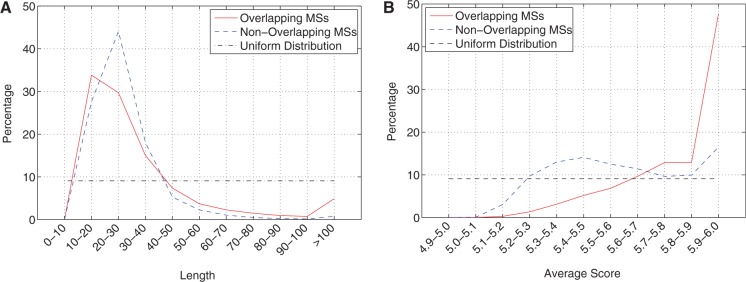
The distributions of the length (**A**) and average score (**B**) of two groups of MSs detected in the human chromosome 19. The first group consisted of MSs overlapping with MSs located by RepeatMasker or by STAR. MSs located by MsDetector only comprised the second group.

We also studied the properties of the MSs that were newly identified by MsDetector in comparison to those of the MSs overlapping with detections by RepeatMasker or STAR. We asked two questions: Did the newly identified MSs have different length distribution? How different were their average scores from those of the MSs overlapping with the MSs located by RepeatMasker or STAR? Recall that MsDetector converts a nucleotide sequence to a series of scores. Here, the average score refers to the mean of the scores representing an MS detected by MsDetector. Figures 5A and 5B show the length and the average score distributions of the two groups. The Kullback–Leibler divergence (KLD) measure was applied to quantify the divergence of the two group distributions from each other. The KLD of a distribution from itself is zero. The smaller the value of the KLD is, the similar the two distributions are. The distribution of the lengths of the new MSs diverged slightly from that of the MSs also detected by RepeatMasker or by STAR (KLD: 0.09, KLD of a uniform distribution from that of the overlapping group: 1.35). The distribution of the average scores of the new MSs diverged more noticeably from that of the other group (KLD: 0.46, KLD of a uniform distribution from that of the overlapping group: 1.74). The average score distribution of the new MSs had two peaks at 5.4–5.5 and 5.9–6.0. In contrast, the distribution peak of the other group was at average scores of 5.9–6.0. These results show that (i) MsDetector has the ability to identify new MSs; (ii) the distribution of the length of the new MSs is very similar to that of MSs also detected by RepeatMasker or by STAR and (iii) the new MSs are assortments of perfect and approximate MSs.

### Analysis of MSs detected by RepeatMasker but not by MsDetector

**Figure 6.**
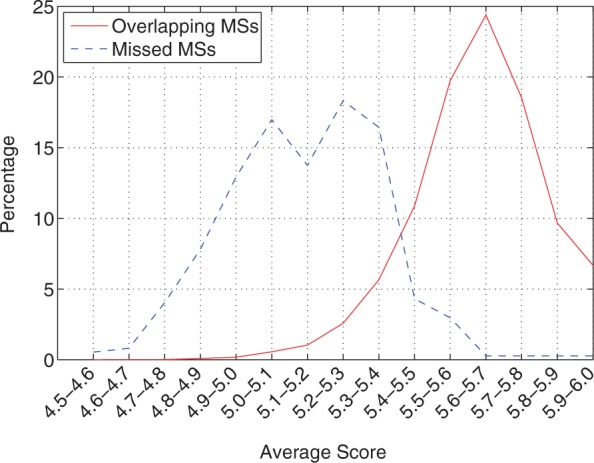
Analysis of the average scores of the MSs identified by RepeatMasker but missed by MsDetector in the human chromosome 19. The first group consisted of the MSs that were detected by RepeatMasker and MsDetector (98%). The second group consisted of the MSs that MsDetector missed (2%).

MsDetector missed 356 (2%) loci detected by RepeatMasker. These loci have almost identical length distribution to those that overlapped with the MSs detected by MsDetector (KLD: 0.09). However, these 356 loci have lower average scores in general. The distribution of the average scores of the missed loci is evidently different from that of the MSs that were missed by MsDetector (Figure 6). Therefore, MsDetector missed RepeatMasker detections that were severely degenerate.

## DISCUSSION

In this section, we compare MsDetector with another HMM-based tool for MSs detection. Then, we discuss future research directions.

### Comparison to closely related work

Tantan is another HMM-based tool to detect MSs. MsDetector differs from Tantan in three main aspects. First, the parameters of the default version of MsDetector were optimized on a human chromosome. We demonstrated the applicability of the default version to other species. In additions, the users can apply the automated pipeline to generate parameters specific to a species of interest. On the other hand, Tantan requires several parameters that the user needs to adjust. Second, the HMM of MsDetector consists of two states, whereas the HMM of Tantan consists of eight states. Third, Tantan does not have an independent filtering component. It uses the HMM to obtain a posterior probability of each nucleotide to belong to an MS segment. Nucleotides that have posterior probabilities of 0.5 or greater are considered MSs. In comparison, MsDetector has a GLM-based filter which is independent of the HMM.

### Future directions

We will consider extending the scoring component by allowing gapped-alignment between words. The current version of MsDetector does not allow gaps while searching for a copy of the word in the flanking sequences. Even though, MsDetector is time-efficient, its running time can be reduced; the algorithm to search for a copy of a word in its vicinity can be further optimized.

## CONCLUSION

We developed a computational tool, MsDetector, to locate perfect and approximate MSs. Our design relies on machine-learning algorithms, specifically HMM and GLM. The main advantage of MsDetector is that all its parameters were optimized. In addition, we provide an automated pipeline to generate parameters specific to a given species. In either case, the user is not obligated to tweak the parameters manually. The results of our evaluations show the following. First, MsDetector located the majority of those MSs found by RepeatMasker as well as STAR in the human chromosome 19 and chromosomes of other five species. Second, MsDetector is time-efficient. Third, MsDetector has a very low FPR. Fourth, our tool is capable of locating new MSs. These four features demonstrate that MsDetector can detect MSs accurately and efficiently in several species advancing the state-of-the-art toward a standard tool.

## SUPPLEMENTARY DATA

Supplementary Data are available at NAR Online: Supplementary Datasets 1–7.

## FUNDING

Funding for open access charge: The Intramural Research Program of the National Institutes of Health, National Library of Medicine.

*Conflict of interest statement*. None declared.

## Supplementary Material

Supplementary Data

## References

[1] Lerat E (2010). Identifying repeats and transposable elements in sequenced genomes: how to find your way through the dense forest of programs. Heredity.

[2] Verstrepen KJ, Jansen A, Lewitter F, Fink GR (2005). Intragenic tandem repeats generate functional variability. Nat. Genet..

[3] Meloni R, Albanése V, Ravassard P, Treilhou F, Mallet J (1998). A tetranucleotide polymorphic microsatellite, located in the first intron of the tyrosine hydroxylase gene, acts as a transcription regulatory element in vitro. Hum. Mol. Genet..

[4] Ramchandran R, Bengra C, Whitney B, Lanclos K, Tuan D (2000). A (GATA)7 motif located in the 5′ boundary area of the human beta-globin locus control region exhibits silencer activity in erythroid cells. Am. J. Hematol..

[5] Boeva V, Regnier M, Papatsenko D, Makeev V (2006). Short fuzzy tandem repeats in genomic sequences, identification, and possible role in regulation of gene expression. Bioinformatics.

[6] Kolpakov R, Bana G, Kucherov G (2003). mreps: efficient and flexible detection of tandem repeats in DNA. Nucleic Acids Res..

[7] Majewski J, Ott J (2000). GT repeats are associated with recombination on human chromosome 22. Genome Res..

[8] Thibodeau SN, Bren G, Schaid D (1993). Microsatellite instability in cancer of the proximal colon. Science.

[9] Richards RI, Holman K, Yu S, Sutherland GR (1993). Fragile X syndrome unstable element, p(CCG)n, and other simple tandem repeat sequences are binding sites for specific nuclear proteins. Hum. Mol. Genet..

[10] Warren ST (1996). The molecular basis of Fragile X syndrome. Science.

[11] Caskey CT, Pizzuti A, Fu YH, Fenwick RGJ, Nelson DL (1992). Triplet repeat mutations in human disease. Science.

[12] Mitas M (1997). Trinucleotide repeats associated with human disease. Nucleic Acids Res..

[13] Ellegren H (2004). Microsatellites: simple sequences with complex evolution. Nat. Rev. Genet..

[14] Frith MC (2011). A new repeat-masking method enables specific detection of homologous sequences. Nucleic Acids Res..

[15] Kurtz S, Choudhuri JV, Ohlebusch E, Schleiermacher C, Stoye J, Giegerich R (2001). REPuter: the manifold applications of repeat analysis on a genomic scale. Nucleic Acids Res..

[16] Edgar RC, Myers EW (2005). PILER: identification and classification of genomic repeats. Bioinformatics.

[17] Achaz G, Boyer F, Rocha EPC, Viari A, Coissac E (2007). Repseek, a tool to retrieve approximate repeats from large DNA sequences. Bioinformatics.

[18] Delgrange O, Rivals E (2004). STAR: an algorithm to search for tandem approximate repeats. Bioinformatics.

[19] Castelo AT, Martins W, Gao GR (2002). TROLL—tandem repeat occurrence locator. Bioinformatics.

[20] Sharma D, Issac B, Raghava GPS, Ramaswamy R (2004). Spectral Repeat Finder (SRF): identification of repetitive sequences using Fourier transformation. Bioinformatics.

[21] Morgulis A, Gertz EM, Schäffer AA, Agarwala R (2006). WindowMasker: window-based masker for sequenced genomes. Bioinformatics.

[22] Du L, Zhou H, Yan H (2007). OMWSA: detection of DNA repeats using moving window spectral analysis. Bioinformatics.

[23] Kofler R, Schlötterer C, Lelley T (2007). SciRoKo: a new tool for whole genome microsatellite search and investigation. Bioinformatics.

[24] Mudunuri SB, Nagarajaram HA (2007). IMEx: imperfect microsatellite extractor. Bioinformatics.

[25] Sokol D, Benson G, Tojeira J (2007). Tandem repeats over the edit distance. Bioinformatics.

[26] Pokrzywa R, Polanski A (2010). BWtrs: a tool for searching for tandem repeats in DNA sequences based on the Burrows–Wheeler transform. Genomics.

[27] Sharma PC, Grover A, Kahl G (2007). Mining microsatellites in eukaryotic genomes. Trends Biotechnol..

[28] Merkel A, Gemmell N (2008). Detecting short tandem repeats from genome data: opening the software black box. Brief Bioinform..

[29] Leclercq S, Rivals E, Jarne P (2007). Detecting microsatellites within genomes: significant variation among algorithms. BMC Bioinformatics.

[30] Bishop CM (1995). Neural Networks for Pattern Recognition.

[31] Rabiner LR (1989). A tutorial on hidden Markov models and selected applications in speech recognition. Proceedings of the IEEE.

[32] Sand A, Pedersen C, Mailund T, Brask A (September, 2010). HMMlib: a C++ library for general hidden Markov models exploiting modern CPUs. The Ninth International Workshop on Parallel and Distributed Methods in Verification.

[33] Nabney IT (2002). NETLAB: Algorithms for Pattern Recognition.

[34] Benson G (1999). Tandem repeats finder: a program to analyze DNA sequences. Nucleic Acids Res..

[35] Saha S, Bridges S, Magbanua ZV, Peterson DG (2008). Empirical comparison of ab initio repeat finding programs. Nucleic Acids Res..

[36] Schneider TD, Stephens R (1990). Sequence logos: a new way to display consensus sequences. Nucleic Acids Res..

