# Supplementary material for: MsDetector: toward a standard computational tool for DNA microsatellites detection
Source: Nucleic Acids Res. 2012 Oct 2;41(1):e22. doi: 10.1093/nar/gks881 (PMC3592430; doi:10.1093/nar/gks881)
Supplement: Supplementary Data [file supp_41_1_e22__index.html]

MsDetector: toward a standard computational tool for DNA microsatellites detection — MsDetector: toward a standard computational tool for DNA microsatellites detection — Supplementary Data 

# MsDetector: toward a standard computational tool for DNA microsatellites detection

## Supplementary Data

files

**Files in this Data Supplement:**

- Supplementary Data - gz file
- Supplementary Data - gz file
- Supplementary Data - gz file
- Supplementary Data - gz file
- Supplementary Data - gz file
- Supplementary Data - gz file
- Supplementary Data - gz file
